# Quality Assessment of Wheat Bread Incorporating Chia Seeds

**DOI:** 10.3390/foods10102376

**Published:** 2021-10-07

**Authors:** Greta Adamczyk, Eva Ivanišová, Joanna Kaszuba, Inna Bobel, Kateryna Khvostenko, Michał Chmiel, Nataliia Falendysh

**Affiliations:** 1 Department of Food Technology and Human Nutrition, Institute of Food Technology and Nutrition, College of Natural Sciences, University of Rzeszow, Zelwerowicza Street 4, 35-601 Rzeszów, Poland; jkaszuba@ur.edu.pl; 2 Institute of Food Sciences, Faculty of Biotechnology and Food Sciences, Slovak University of Agriculture in Nitra, Trieda Andreja Hlinku 2, 949 76 Nitra, Slovakia; eva.ivanisova@uniag.sk; 3 Department of Bakery and Confectionary Goods Technologies, Educational and Scientific Institute of Food Technology, National University of Food Technologies, 68 Volodymyrska Street, 01601 Kyiv, Ukraine; inna_3@ukr.net (I.B.); falen@nash.net.ua (N.F.); 4 Department of Bread, Confectionary, Pasta Products and Food Concentrates Technology, Odessa National Academy of Food Technologies, 112 Kanatna Street, 65039 Odessa, Ukraine; katekhvostenko@gmail.com; 5 Department of Carbohydrates Technology, Faculty of Food Technology, University of Agriculture in Kraków, Balicka Street 122, 30-149 Kraków, Poland; michops85@gmail.com

**Keywords:** wheat bread, chia seeds, the single-stage method, nutritive value

## Abstract

The aim of this study was to develop a concept of production for wheat bread enriched with chia seeds and to examine selected physicochemical properties. The examined product was wheat-flour bread made by a single-phase method, using yeast. The production concept assumed the modification of the recipe by replacing part of the wheat flour (1 or 5% *w*/*w*) with whole (CHw) or ground chia seeds (CHg). Bread quality was determined by calculating: dough yield, bread yield, baking loss, total baking loss and loaf volumes. Color was determined using the CIE L*a*b* system. In the texture analysis, the following texture parameters were measured: hardness, cohesion, chewiness and elasticity. The contents of crude fat, crude fiber, total protein, total ash and the total content of polyphenols were assessed to characterize the nutritive value of the bread. The breads with 1% addition of chia (1%CHw, 1%CHg) were characterized by the highest volume of loaves, which increased by at least 8.6% compared to the control bread (C), while in the case of 5% chia, the loaf volume depended on the form of seeds (better results were obtained with whole seeds). Substituting wheat flour with 1% chia seeds (whole or ground) resulted in a significant increase in nutritional value. For potential bread manufacturers, from a technological and economic point of view, replacing wheat flour with whole chia seeds at 1% (*w*/*w*) is most advantageous, compared to 5% (*w*/*w*).

## 1. Introduction

As the nutritional awareness of consumers increases and changes are observed in their lifestyle and shopping habits, food producers are constantly improving products already on the market, or searching for new solutions and food products. To increase the nutritive and health value of their products, many producers are enriching such products with additives from the “superfood” group. Superfoods, or functional foods, are a group of products that contain significant amounts of nutrients that are beneficial for the body, such as: phytochemicals, vitamins, enzymes, essential amino acids, minerals and other bioactive substances [1].

Bread is one of the most popular products in the daily diet of people around the world. In Poland, bread occupies the third place in the daily diet, right after dairy products and potatoes [2,3]. Bread is made by combining flour, water, salt and leaven or yeast. Bakers, in order to make their products more attractive, increase their nutritive value and taste-aroma features, often, in addition to the basic ingredients, by using various types of additives. These include, for example, eggs, dairy products, lecithin, various types of flour and seeds (poppy, pumpkin, sunflower, flax, cumin, chia) and others. Some of these can be classified as functional foods [4]. 

Chia (*Salvia hispanica* L.) comes from areas in Central America (Guatemala and Mexico), but is presently also cultivated in Argentina, Australia, Colombia, Bolivia, Peru, Europe, and America [5]. The high nutritional value of chia seeds is a result of the contents of fat (30–34%), protein (15–26%), carbohydrates (26–41%) and dietary fiber (18–30%) [6,7]. The content of individual components depends on the variety as well as the environmental and growing conditions [8]. Chia seeds are characterized by a high content of α-linolenic acid (ALA), belonging to *n*-3 polyunsaturated fatty acids (PUFA), with a small amount of linoleic acid (LA), which belongs to *n*-6 PUFA. The content of essential fatty acids such as ALA and LA determines the unique role of the seeds of this plant in human nutrition. Both of these fatty acids must be provided by foods, since they cannot be synthesized in the body. Chia seeds also contain a high biological value of protein, and amino acids such as isoleucine, leucine, lysine, and valine [8,9,10,11]. Chia seeds have a high health-promoting potential, exerting hypolipemic, hypoglycemic, antimicrobial, and also immunostimulant effects. The antioxidant properties of these seeds are also crucial, as they contain natural antioxidants such as sterols, tocopherols, carotenoids and phenolic compounds, which can quench and scavenge free radicals and cation radicals [12,13,14,15].

Several studies have shown that chia seeds can be used for production of bread, and that it contributes to the technological quality and texture parameters, depending on the method of preparation or additive type (flour or seeds) [13,16,17,18,19].

Attention should be paid to the fact that the manufacturers would like to obtain a good price for the finished product. Chia seeds are an expensive raw material, and when grounded, they are also unstable due to the content of unsaturated fat, resulting from oxidative changes of fats in material. The best solution for manufacturers would be to use a small addition of chia seeds, which is their most stable form for storage, and which would have a positive effect on the nutritional value and parameters of the final product. In this study, wheat flour was substituted with chia seeds in the amounts of 1% and 5% (*w*/*w*), and additionally, whole or ground seeds were used. In the literature, the most common substitution of flour with chia seeds ranges from 2.5 to 7.5%, and values up to 10% have been used [13,18,20,21]. Some authors have shown that wheat breads with 7.5% chia seeds are not desirable to consumers [18]; hence, a limit of 5% was adopted in our work. Therefore, there is no information in the literature on the minimum replacement of wheat flour with chia seeds, which would be beneficial for potential manufacturers (low cost of raw materials) while at the same time increasing the nutritional value of the bread and the appearance of the final product from a technological and organoleptic point of view.

Firstly, the aim of our work was to show to potential manufacturers that the small substitution amount of wheat flour with chia seeds in the wheat bread formulation may have a positive effect on its technological parameters, improve its nutritional value and minimize changes in color of the product. Secondly, we wanted to show the differences in bread quality parameters in the case of replacing wheat flour with whole or ground chia seeds in the presented bread formulation.

## 2. Materials and Methods

### 2.1. Materials

Raw materials for the breads were purchased from a local hypermarket in Rzeszów (Poland). These were: chia seeds, producer Casa Del Sur (Peru); wheat flour type 650, producer: PZZ KAPKA (Poland); baker’s yeast (*Saccharomyces cerevisiae*), manufacturer Lellemand; salt from the Wieliczka Salt Mine, producer Kotánya; and tap water.

### 2.2. Sample Preparation

The examined material was wheat bread made according to the direct method (the single-phase method), ICC standard No. 131 [20], with own modifications. The dough, obtained by mixing wheat flour (type 650), yeast, salt, water (in an amount of mL corresponding to the water absorption of flour for a dough consistency of 350 FU) and in the recipe of bread with the addition of chia seeds (CH). Either whole (w) or ground (g) chia seeds (according to the recipes presented in Table 1) were then kneaded using a laboratory kneader (Mesko AGD, Poland). The water absorption of flour was determined using a farinograph-E (Brabender, Germany) according to ICC Standard No. 115/1 [20].

The analysis involved adding whole or ground chia seeds as a substitute to the wheat flour in amounts of 1% or 5% in relation to the flour by weight (1%CHw, 1%CHg, 5%CHw, 5%CHg). The control sample (C) was wheat bread made from wheat flour without the addition of chia seeds.

After mixing and kneading all ingredients, dough underwent a 30-min fermentation process at 30 °C and a relative humidity of 75–80% in a proofing chamber (Sveba-Dahlén, Sweden). The dough was then punched by hand and fermented again for 30 min. Afterwards, chunks of about 250 g were formed, and were left for optimal proofing of the dough, and finally were baked at 230 °C for 30 min in an electric oven (Sveba-Dahlén, Sweden). 

### 2.3. Determination of Quality Indicators of the Baking Process and Product 

In the obtained wheat bread and in the breads with ground or whole chia seeds, the following baking quality indicators were determined: dough yield (%), baking loss (%), total baking loss (%) and bread yield (%). Baking loss (%) was calculated as ([the weight of the dough formed for baking—the weight of the hot bread]/[the weight of the dough formed for baking] × 100) and total baking loss (%) was calculated as ([the weight of the dough formed for baking—the weight of the bread after cooling]/[the weight of the dough formed for baking] × 100) [21]. 

Dough chunks, ready-made loaves of bread and the breads cooled down after 24 h were weighed [22]. The volume of bread was determined with the Sa-Way apparatus (Sadkiewicz Instruments, Poland) according to AACC Method No. 10–05.01 [23]. Bread specific volume was calculated as the ratio of the volume to weight. 

The determinations were made in two repetitions.

### 2.4. Nutritional Assessment of Breads

The prepared breads were dried with a freeze-drying unit ALPHA 1-2 LD plus (Martin Christ Gefriertrocknungsanlagen GmbH, Osterode am Harz, Germany), and then the samples were ground. In the bread samples were determined nutritional parameters. Total ash content was measured in accordance with ICC Standard No. 104/1 [20] in a muffle furnace (Neberterm, Germany). Total nitrogen was determined by Kjeltec System (Foss, Hilleroed, Denmark) and converted into total protein content using the *N* × 6.25 conversion factor based on AACC Method No. 46-11.02 [23]. Crude fat content in the examined breads was detected by means of the extraction-weight method, using a Soxtec 2050 (Foss, Hilleroed, Denmark) apparatus was determined by AACC Method No. 30-10.01 [23]. Crude fiber content was assessed by an Ancom 200 Fiber Analyzer (Ancom Technology, Macedon, NY, USA). In this procedure, a 1g sample (calculated per dry matter) was weighed (with accuracy up to 0.0001 g) in a special filter bag, defatted with petroleum ether, air-dried and placed into the analyzer. Then, 2000 mL of 0.1 M sulphuric acid was added, and the samples were hydrolyzed at 100 °C for 45 min. After completing this process, the samples were washed with hot distilled water 3 times. The water was gently pressed from the bags and the samples were air-dried and put into a laboratory drier at 105 °C (WTB, Binder, Germany) for 2 h. After cooling to room temperature, the samples were re-weighed and incinerated at 550 °C for 2 h, in pre-weighed crucibles. The incinerated crucibles were then weighed. Crude fiber content (%) was calculated using the following equation:

(1) $$X\;\left(\%\right)=\frac{c-\left(a\times d\right)}{b}\times 100$$

where:

a—bag weight [g],

b—weight of the sample [g],

c—weight loss of the sample [g],

d—coefficient.

All parameters were determined in three replications for each type of bread. 

### 2.5. Texture Parameters

Crumb texture profile analysis (TPA) was performed using a Brookfield CT3 texture analyzer (USA) equipped with Texture Pro CT V1.2 Build 9 software. Cylindrical crumb samples (h = 27 mm, d = 27 mm, V = 22 cm^3^) from different parts of the loaves were subjected to a compression test using a 25-mm-diameter stainless steel disc. It was compressed twice along its axis at a speed of 50 mm·s^−1^ until 50% deformation and a pause of 5 s were obtained.

The following texture parameters were determined: hardness (N), cohesiveness (−), elasticity (−), chewiness (mJ) and springiness (mm) [22]. All parameters were determined in three replications, for each type of bread. 

### 2.6. Color Analysis

Color analysis was conducted using a spectrophotometer (HunterLab, Reston, VA, USA), based on the CIE L*a*b* system. The following parameters were determined: lightness (L*), redness+/greenness (a*), and yellowness+/blueness (b*). Arithmetic means from three repetitions were calculated for each type of wheat bread [24]. 

### 2.7. Total Polyphenol Content

Total polyphenol content (TPC) was measured spectrophotometrically, using the modified Folin–Ciocalteu method as described by Singleton and Rossi (1965) [25]. For this analysis, extracts from freeze-dried samples were made. From each type of bread, 0.5 g was weighed on an analytical balance (with accuracy up to 0.0001 g) before adding 20 mL of 70% methanol solution. The properly prepared suspensions were placed in an ultrasonic bath, shaken at 20 °C for 20 min, and centrifuged in a laboratory centrifuge for 5 min (7000 rpm). A total of 2 mL distilled water, 0.2 mL Folin–Ciocalteu reagent and 1 mL 20% Na_2_CO_3_ solution (*m*/*m*) were added to 0.1 mL of the extract in spectrophotometric cuvettes. The samples were then left for 1 h. Absorbance was measured at λ = 765 nm against distilled water. Gallic acid was used as a standard and the results were expressed in mg gallic acid equivalent (GAE) per 1 g d.m. (dry matter) of sample. TPC was determined in three replications, for each type of bread. 

### 2.8. Statistics

Statistical analysis was performed using Statistica 13.3 (TIBCO Software Inc., Palo Alto, CA, USA) including one-way ANOVA. To establish the significance of the differences between the average values, Duncan’s test was performed (*p* < 0.05).

## 3. Results and Discussion

Table 2 presents the results obtained for the yield of dough and bread as well as the values of baking loss and total baking loss.

The obtained results showed that the enrichment of wheat bread with whole or ground chia seeds affected the dough yield. An approximately 2% decrease was observed in the dough yield in bread with a 1% addition of whole and ground chia seeds (1%CHw and 1%CHg) compared to the control bread (C), while about a 2% increase was noted in this parameter in the breads with 5% addition of chia seeds (5%CHw and 5%CHg). There were no differences in the values between the samples 1%CHw and 1%CHg, but statistically significant differences were observed between those coded as 5%CHw and 5%CHg, which proves that there was a correlation between the addition of 5% ground chia seeds and dough yield. In turn, statistically significant differences were found with regard to the proportion of chia seeds (1% and 5% *w*/*w* respectively) and the control sample (C). The findings of Kaszuba et al. (2017) [26] showed that the addition of oil plant seeds to flour affects the dough yield. The yields of the breads 5%CHw and 5%CHg decreased by an average of 2% compared to the control sample, while 1%CHw and 1%CHg samples decreased by about 3%. Romankiewicz et al. (2017) [13] claimed that the addition of chia seeds (from 2 to 8%) to bread reduced its yield as well. Chia seeds, due to their high content of fiber and mucilage gel, retain water and prevent its evaporation during heat treatment. In the present study, the form in which chia seeds were added (whole or ground), had no significant effect on the baking loss of the examined wheat breads but total baking loss increased with the addition of chia seeds. Romankiewicz et al. (2017) [13] observed the same dependence of total baking loss on the addition of chia seeds in amounts from 2 to 4%. According to Steffolani at al. (2014) [27], Zettel et al. (2014) [28] and Romankiewicz et al. (2017) [13], the addition of 6% and 8% chia significantly reduced the value of the baking loss of breads. Dietary fiber and mucilage in chia seeds combines free water in dough and prevents its evaporation during the process of baking [28].

Table 3 contains the values of the specific volume and the volume of the loaves of the tested wheat bread.

It was found that the 1% addition of whole (1%CHw) and ground (1%CHg) chia seeds led to an increase in bread volume compared to the control sample (C). There were also statistically significant differences between the amounts of chia seeds added (1% and 5% *w*/*w*). As a result of the increased addition of ground seeds (up to 5%), the specific volume of the loaves obtained was similar to the control sample. In turn, adding 5% ground seeds (5%CHg) resulted in a slight decrease in the bread volume (545 cm^3^) compared to the loaves containing 5% whole chia seeds (580 cm^3^). Huerta et al. (2018) [29] showed the potential of chia seeds as a substitute gum in gluten-free breads. Their study on the specific volume in free-gluten breads indicated significantly lower values in the case of higher amounts of chia seeds (5 and 7.5%) and not significant differences in volume in the amount of 2.5% chia compared to the control sample. Similar results were obtained by Kowalski et al. (2020) [18]. They substituted wheat flour with 2.5, 5.0, or 7.5% of ground chia seeds and observed a decrease in bread volume (from 898 to 883 cm^3^), but the results were not statistically significantly different. According to Sharma and Chauhan (2000) [30], the formation of a protein–lipid complex hinders the formation and retention of gas, which results in a reduced bread volume. The reduction in bread volume may be due to the introduction of fiber-rich products into the system. The presence of fiber in the dough negatively affects the formation and properties of gluten, and this reduces its ability to retain gases [31,32,33].

The results obtained for the chemical ingredients content and polyphenol content in wheat bread with and without chia seeds are given in Table 4.

In the examined samples, the crude fat content changed depending on the amounts of added chia seeds. The lowest was in the control sample (2.63 g), while in the remaining bread samples, the addition of chia seeds caused an increase in the crude fat content. Of the examined breads containing chia seeds, the 1%CHg loaf had the lowest crude fat content, while breads 1%CHw and 5%CHw were statistically similar with regard to this parameter.

As a result of replacing a portion of the flour with chia seeds, an increase was observed in the crude fiber content in the examined samples, except for the 1%CHw sample, where this value slightly decreased.

The results obtained for the total protein content in the examined breads, presented in Table 4, indicate that its content increased after adding chia seeds, compared to the control sample. An increase in the amount of total protein content in bread was also noted after increasing the proportion of chia seeds from 1% to 5%. The findings of Pastuszka et al. (2012) [34] showed an increase in the content of protein, fat and dietary fiber in gluten-free bread as a result of adding linseed oil.

This study demonstrated that partial replacement of flour by chia seeds influences the content of polyphenols in the examined wheat breads. As shown in Table 4, the quantity of added seeds had an effect on the content of polyphenolic compounds in the final product and increased with an increase in the proportion of the seeds. The amount of polyphenols in the control sample was 0.09 mg GAE/g d.m. The 1% addition of chia seeds to the bread baking blend slightly elevated the content of polyphenolic compounds in the final products, up to 0.12 and 0.10 mg GAE/g d.m, in samples 1%CHg and 1%CHw, respectively. The increased proportion of added seeds caused an increase in the content of polyphenols of, respectively, 0.17 and 0.13 mg GAE/g d.m in the 5%CHg and 5%CHw samples. 

The texture profile analysis parameters of the wheat breads enriched with chia seeds and the control sample are depicted in Table 5. The most frequently determined parameter in bread crumb texture is the hardness (N), because it describes the maximum force during the first bite [35]. The presented control bread (without chia seeds addition) had crumbs with a hardness of 9.14 N. A significant reduction of this parameter was observed in 1%CHw and 1%CHg (6.55 and 6.71 N, respectively) and a slight but not significant reduction in hardness was noticed in 5%CHw and 5%CHw (8.56 and 8.22 N). Samples 1%CHw and 1%CHg had higher volume than other samples, so hardness was lower, while 5%CHw and 5%CHw were characterized by smaller loaf volumes (Table 3), which determines their greater hardness. According to Romankiewicz et al. (2017) [13], the presence of chia seeds in bread weakens the gluten matrix, causing the weakening of the crumb cell structure and thus resulting in the obtained crumbs having lower volumes. Moreover, the chemical composition of bread with chia seeds had more fat, and this may be due to the decrease in bread hardness. An additional component of chia seeds is mucilage gel, which probably caused a decrease of bread hardness.

The springiness of the bread crumb describes the return of the crumbs to their original shape following compression. The cohesiveness parameter is determined on the work during the first and second compressing [35]. The obtained results (Table 5) showed statistical significance in the springiness parameter and not statistically significant differences in cohesiveness compared to the control bread. The results of crumb elasticity for all of the examined breads were similar to the control sample.

With regard to the chewiness, the C, 5%CHw, and 5%CHg samples did not differ significantly. On the other hand, the samples with 1% addition of whole or ground chia seeds were statistically similar to each other and showed lower chewiness. The control sample exhibited the highest chewiness (76.95 mJ), while the lowest (54.95 mJ) was noted in the sample with 1% addition of ground chia seeds. The lower hardness of the crumbs (6.55 and 6.71 N) meant that the chewiness also assumed lower values (55.40 and 54.95 mJ) (Table 5), because higher loaf volumes were obtained (Table 3). A study by Swami et al. (2015) [36] proved that adding chia reduces the texture quality by weakening the structure of the bread crumb due to the chemical composition of chia seeds, mainly the high content of fat. They also found that the addition of chia seeds had no significant effect on the cohesiveness and elasticity of the bread.

Table 6 presents the values of the color parameters (L*, a*, and b*) of the wheat bread with and without the addition of chia seeds.

Our study revealed that the crumb color of the bread containing chia seeds was darker compared to the control sample, and the differences were statistically significant (*p* < 0.05). An increase in the content of chia seeds from 1% to 5% lowered the value of the lightness parameter (L*). The sample with the highest proportion of ground chia seeds (5%CHg) was the darkest (59.11). Bread with the same amount of seeds but with whole seeds (5%CHw) was about 5% lighter (62.51). Ground chia seeds, but in a lower proportion (1%CHg) gave the same effect in the lightness of the crumbs (62.43) as the whole seeds—62.51 (5%CHw). Coelho and Sallas-Mellado (2015) [16] proved the mutual dependence of the addition of chia seeds and the darkening of the bread color, due to the increased content of the seed-derived fiber and fat. In the samples with added whole chia seeds (1%CHw, 5%CHw), the values of parameter a* did not differ statistically; in the remaining ones, the differences were higher and significant. The value of parameter b* was the highest in the control sample (18.73) and the lowest in the 5%CHg (13.65). All of these results differed statistically. During the baking process, the temperature in the bread crumbs is about 100 °C, so the color of the crumbs is mainly dependent on the materials used. The breads with whole or ground chia seeds had chia seeds pigments, which contains phenolic compounds (e.g., chlorogenic acid, caffeic acid, ferulic acid, quercetin and other), giving color to the seeds [27,37,38]. Similarly, Coelho and Salas-Mellado (2015) [16], Romankiewicz et al. (2017) [13] and Huerta et al. (2018) [29] obtained darker breads than the standard, when preparing breads with chia seeds flour.

## 4. Conclusions

The 1%CHw and 1%CHg samples were characterized by the highest volume of loaves, which increased by at least 8.6% compared to the control bread (C), while in the case of 5%CHw and 5%CHg, the loaf volume depended on the form of the seeds (with better results being obtained with whole seeds). The presence of 5% ground chia seeds in bread weakens the gluten matrix and causes the weakening of the crumb structure. Therefore, lower bread volumes were obtained (from 562.50 to 545.00 cm^3^). Moreover, the reduction in bread volume may be due to the fact that the presence of fiber in the dough negatively affects the formation and properties of gluten, and this reduces its ability to retain gases.

The bread with chia seeds, which contained a higher content of fat compared to the control, as well as the presence of mucilage gel chia, may cause a decrease in the hardness of the bread crumb. 

Fortification of wheat bread with oilseeds with a high antioxidant potential is a desirable alternative to recipe modification, both in terms of nutritional value and the quality of the obtained bread. An important finding in these studies is the fact that the value of the addition of oilseeds to bread depends on the form in which it is applied to the product. Grounded chia seeds, compared to whole seeds, increased some nutritional values (fiber, polyphenol content) of the finished product to a greater extent.

In conclusion, we found that for potential bread manufacturers, from a technological and economic point of view, replacing wheat flour with whole chia seeds at 1% (*w*/*w*) is most advantageous, compared to 5% (*w*/*w*). Despite such a small substitution of wheat flour with oilseeds, good-quality bread was obtained. 

## Figures and Tables

**Table 1:** Recipes used for the production of wheat bread and wheat breads with chia seeds by the single-phase method.

Sample	Chia Seeds (g)	Wheat Flour (g)	Water (ml)	Yeast (g)	Salt (g)
C	0	320.00	202.70	9.6	3.20
1%CHw	3.20	316.80	202.70	9.6	3.20
1%CHg	3.20	316.80	202.70	9.6	3.20
5%CHw	16.00	304.00	202.70	9.6	3.20
5%CHg	16.00	304.00	202.70	9.6	3.20

C—control sample—wheat bread without chia; 1%CHw—wheat bread with 1% whole chia seeds; 1%CHg—wheat bread with 1% ground chia seeds; 5%CHw—wheat bread with 5% whole chia seeds; 5%CHg—wheat bread with 5% ground chia seeds.

**Table 2:** Baking parameters of wheat bread with and without chia seeds.

Sample	Dough Yield (%)	Baking Loss (%)	Total Baking Loss (%)	Bread Yield (%)
C	168.10 ± 0.41 ^b^	8.04 ± 1.26 ^a^	14.45 ± 1.01 ^a^	149.12 ± 1.75 ^b^
1%CHw	163.64 ± 0.97 ^a^	10.18 ± 0.98 ^a^	16.63 ± 0.22 ^b^	145.32 ± 0.39 ^a^
1%CHg	165.51 ± 1.03 ^a^	9.08 ± 1.48 ^a^	15.90 ± 0.97 ^ab^	146.58 ± 1.69 ^ab^
5%CHw	171.05 ± 1.25 ^c^	8.71 ± 1.24 ^a^	15.54 ± 0.77 ^ab^	147.21 ± 1.35 ^ab^
5%CHg	174.87 ± 1.87 ^d^	8.87 ± 0.80 ^a^	15.13 ± 0.39 ^ab^	147.93 ± 0.67 ^ab^

The values correspond to the mean values ± standard deviation. Parameters marked with the same letter in the column do not differ significantly at a confidence level of α = 0.05.

**Table 3:** Specific volume and loaf volume of wheat bread with and without chia seeds.

Sample	Specific Volume (cm^3^/g)	Loaf Volume (cm^3^)
C	2.62 ± 0.05 ^a^	562.50 ± 17.68 ^ab^
1%CHw	2.98 ± 0.07 ^b^	621.00 ± 12.73 ^d^
1%CHg	2.90 ± 0.03 ^b^	611.00 ± 1.41 ^cd^
5%CHw	2.75 ± 0.04 ^c^	580.00 ± 14.14 ^bc^
5%CHg	2.57 ± 0.05 ^a^	545.00 ± 7.07 ^a^

The values correspond to the mean values ± standard deviation. Parameters marked with the same letter in the column do not differ significantly at a confidence level of α = 0.05.

**Table 4:** Content of crude fat, crude fiber, protein, ash and total polyphenols content in the examined wheat bread with and without chia seeds.

Sample	Crude Fat Content (%)	Crude Fibre Content (%)	Total Protein Content (%)	Total Ash Conent (%)	Total Polyphenol Content (mg GAE/100 g d.m.)
C	2.63 ± 0.13 ^b^	0.48 ± 0.00 ^ac^	9.49 ± 0.28 ^c^	1.30 ± 0.15 ^b^	0.09 ± 0.01 ^a^
1%CHw	3.82 ± 0.65 ^a^	0.47 ± 0.01 ^c^	10.28 ± 0.16 ^ab^	1.26 ± 0.27 ^b^	0.10 ± 0.01 ^a^
1%CHg	3.36 ± 0.36 ^ab^	0.51 ± 0.00 ^ab^	10.17 ± 0.07 ^a^	0.72 ± 0.41 ^a^	0.12 ± 0.04 ^ab^
5%CHw	4.10 ± 0.26 ^a^	0.51 ± 0.00 ^b^	10.73 ± 0.14 ^b^	1.19 ± 0.28 ^ab^	0.13 ± 0.00 ^ab^
5%CHg	3.62 ± 0.08 ^a^	0.50 ± 0.02 ^ab^	10.58 ± 0.21 ^ab^	1.13 ± 0.09 ^ab^	0.17 ± 0.00 ^b^

The values correspond to the mean values ± standard deviation. Parameters marked with the same letter in the column do not differ significantly at a confidence level of α = 0.05.

**Table 5:** Texture parameters of wheat bread without and with the addition of chia seeds (TPA test).

Sample	Hardness [N]	Cohesiveness (−)	Elasticity (−)	Chewiness (mJ)	Springiness (mm)
C	9.14 ± 0.18 ^a^	0.66 ± 0.01 ^a^	0.40 ± 0.02 ^ab^	76.95 ± 0.78 ^a^	12.87 ± 0.06 ^a^
1%CHw	6.55 ± 0.36 ^b^	0.65 ± 0.03 ^a^	0.39 ± 0.01 ^ab^	55.40 ± 1.13 ^b^	13.00 ± 0.13 ^ac^
1%CHg	6.71 ± 0.21 ^b^	0.64 ± 0.00 ^a^	0.39 ± 0.00 ^ab^	54.95 ± 1.06 ^b^	12.80 ± 0.01 ^ab^
5%CHw	8.56 ± 0.35 ^a^	0.65 ± 0.01 ^a^	0.41 ± 0.00 ^b^	75.60 ± 2.40 ^a^	12.65 ± 0.08 ^b^
5%CHg	8.22 ± 0.64 ^a^	0.66 ± 0.01 ^a^	0.37 ± 0.00 ^a^	69.85 ± 5.59 ^a^	13.11 ± 0.08 ^c^

The values correspond to the mean values ± standard deviation. Parameters marked with the same letter in the column do not differ significantly at a confidence level of α = 0.05.

**Table 6:** Color parameters of the wheat bread with and without chia seeds.

	Color Parameters		
Sample	L*	a*	b*
C	67.54 ± 0.25 ^d^	1.72 ± 0.07 ^b^	18.73 ± 0.01 ^e^
1%CHw	65.28 ± 0.08 ^c^	1.42 ± 0.01 ^a^	17.24 ± 0.01 ^d^
1%CHg	62.43 ± 0.04 ^b^	1.63 ± 0.04 ^b^	16.40 ± 0.04 ^c^
5%CHw	62.51 ± 0.67 ^b^	1.34 ± 0.04 ^a^	15.77 ± 0.22 ^b^
5%CHg	59.11 ± 0.14 ^a^	2.06 ± 0.02 ^c^	13.65 ± 0.01 ^a^

The values correspond to the mean values ± standard deviation. Parameters marked with the same letter in the column do not differ significantly at a confidence level of α = 0.05.

## Data Availability

Data sharing is not applicable to this article.
